# Transcription factor YcjW controls the emergency H_2_S production in *E. coli*

**DOI:** 10.1038/s41467-019-10785-x

**Published:** 2019-06-28

**Authors:** Lyly Luhachack, Aviram Rasouly, Ilya Shamovsky, Evgeny Nudler

**Affiliations:** 1 Department of Biochemistry and Molecular Pharmacology, New York University School of Medicine, New York, NY 10016 USA; 2 Howard Hughes Medical Institute, New York University School of Medicine, New York, NY 10016 USA

**Keywords:** Biochemistry, Microbiology

## Abstract

Prokaryotes and eukaryotes alike endogenously generate the gaseous molecule hydrogen sulfide (H_2_S). Bacterial H_2_S acts as a cytoprotectant against antibiotics-induced stress and promotes redox homeostasis. In *E. coli*, endogenous H_2_S production is primarily dependent on 3-mercaptopyruvate sulfurtransferase (3MST), encoded by *mstA*. Here, we show that cells lacking 3MST acquire a phenotypic suppressor mutation resulting in compensatory H_2_S production and tolerance to antibiotics and oxidative stress. Using whole genome sequencing, we identified a non-synonymous mutation within an uncharacterized LacI-type transcription factor, *ycjW*. We then mapped regulatory targets of YcjW and discovered it controls the expression of carbohydrate metabolic genes and thiosulfate sulfurtransferase PspE. Induction of *pspE* expression in the suppressor strain provides an alternative mechanism for H_2_S biosynthesis. Our results reveal a complex interaction between carbohydrate metabolism and H_2_S production in bacteria and the role, a hitherto uncharacterized transcription factor, YcjW, plays in linking the two.

## Abstract (web summary)

Hydrogen sulfide (H2S) production in *Escherichia coli* is controlled by the sulfurtransferase 3MST. Here, the authors describe an alternative mechanism for H2S biosynthesis via activation of the thiosulfate sulfurtransferase PspE, a process mediated by the transcription factor YcjW.

## Introduction

The ubiquitous gaseous molecule H_2_S can be generated in many enzymatic pathways and from various substrates, among which L-cysteine is usually the predominant one^1,2^. The main pathway by which *E. coli* generates H_2_S when grown aerobically in nutrient-rich LB is via 3-mercaptopyruvate sulfurtransferase, 3MST, encoded by *mstA*, formerly known as *sseA*^3,4^. Phenotypic consequences of decreased bacterial H_2_S production include greater susceptibility to multiple classes of antibiotics and oxidative stress^3–6^. The H_2_S-mediated resistance against H_2_O_2_ can, in part, be explained by sequestration of ferric iron to diminish damaging Fenton chemistry^4^. Moreover, enzymatic production of H_2_S consumes the excess of intracellular cysteine^4^, which otherwise could behave as a pro-oxidant that fuels the Fenton reaction^7^. Bacterial H_2_S has also been demonstrated recently to be important in protecting pathogens against host innate immune response^8^. Given the overall importance of endogenous H_2_S production for bacterial defense against stress, it has been considered as a promising target for antimicrobial therapy^8,9^. However, to design specific inhibitors of H_2_S production, one has to be aware of alternative endogenous sources of H_2_S, which can be induced in response to inactivation of the main H_2_S enzymatic pathway(s). Here, we describe one such new alternative source of H_2_S production in *E. coli* and the mechanism of its regulation.

## Results and discussion

### Phenotypic suppression of *ΔmstA* depends on alternative mechanism for H_2_S synthesis

**Fig. 1:**
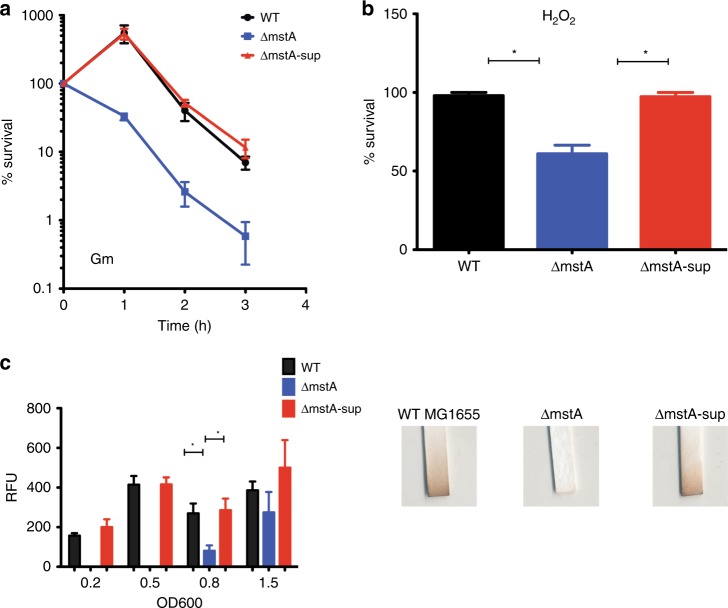
*E. coli* MG1655 lacking 3MSTA acquires phenotypic suppression and has increased H_2_S levels and tolerance to Gm and H_2_O_2_. **a** Δ*mstA-sup* has increased survival rate compared with Δ*mstA* when treated with 2 µg  ml^−1^ gentamicin in a time-kill assay. Values correspond to colony-forming units (c.f.u). **b** Δ*mstA-sup* also has increased tolerance after exposure to 5 mM H_2_O_2_ for 30 min. **c** H_2_S production as measured with fluorescent probe, WSP5. Relative fluorescent units are normalized to OD_600_ and minus the background fluorescent of PBS buffer + 100 µM L-cysteine and WSP5. H_2_S reacts with lead acetate, leading to staining of strips (Sigma-Aldrich). Values are means ± SD (*n* = 3). *p < 0.05 as determined by the Student's *t* test. Source data are provided as a Source Data file

In the course of our work, the antibiotics-sensitive strain *ΔmstA* often reverted to the resistant phenotype of the isogenic parent when challenged with different antibiotics. The *ΔmstA* variant, referred to as *ΔmstA-*sup in this study, was indistinguishable from wild-type in time-kill assay analysis and growth curves of cells exposed to gentamicin, nalidixic acid, and carbenicillin (Fig. 1a; Supplementary Fig. 1). Furthermore, this strain also had increased tolerance to hydrogen peroxide (H_2_O_2_), compared with its still sensitive parent *ΔmstA* strain (Fig. 1b). Using both the classic lead acetate reactivity test for H_2_S detection and a fluorescent-based probe, WSP5^10^, we confirmed that this phenotypic reversion was concurrent with increased H_2_S production, comparable with wild-type (Fig. 1c). In contrast, significant levels of H_2_S remained undetectable in *ΔmstA* till OD_600_ 1.5.

### S258N substitution in the transcription factor YcjW restores both H_2_S production and antibiotic tolerance in *ΔmstA-*sup

**Fig. 2:**
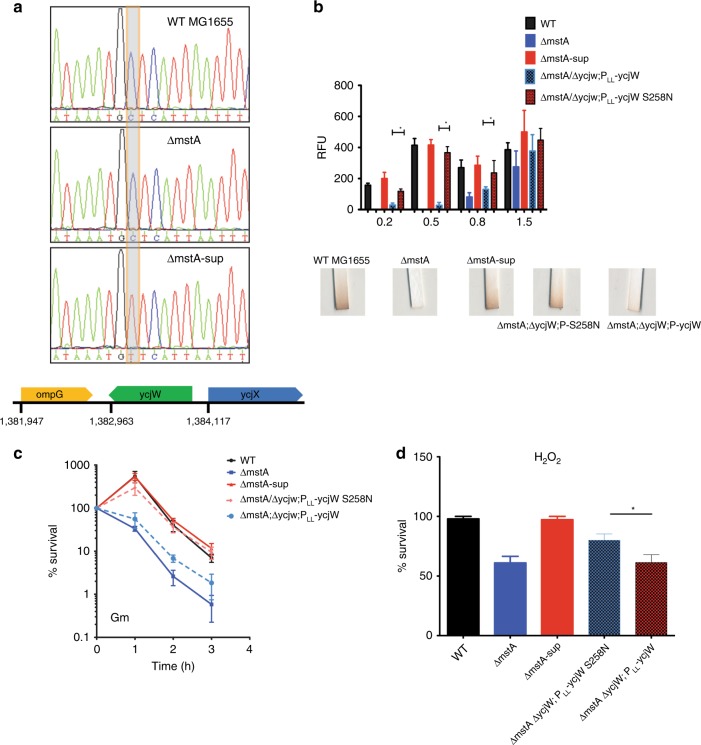
Unique nonsynonymous SNP in Δ*mstA-sup* is mapped to the putative transcription factor *ycjW*. **a** PCR validation of whole-genome sequencing. Displayed are sequences from *E. coli* MG155, Δ*mstA*, and Δ*mstA-sup* of (−) strand. The SNP changes amino acid 258 from serine to asparagine. **b** *ΔmstA***/***ΔycW* strains with plasmid-expressed P_LL_-ycjW or P_LL_-ycjW S258N were measured for H_2_S production. Only *ΔmstA***/***ΔycW*; P_LL_-ycjW S258N had increased H_2_S production with no significant differences with WT or *ΔmstA-sup*. At OD_600_ 1.5, no significant differences exist between strains. **c** Cells exposed to 2 µg ml^−1^ Gm. *ΔmstA***;***ΔycW*; P_LL_-ycjW S258N had increased survival rate. *ΔmstA***/***ΔycW*; P_LL_-ycjW was more sensitive to Gm compared with wild-type and *ΔmstA*-sup. **d** *ΔmstA***/***ΔycW*; P_LL_-ycjW S258N improved tolerance to H_2_O_2_. Values are means ± SD (*n* = 3) for all experiments. *p < 0.05 as determined by the Student's *t* test. Source data are provided as a Source Data file

We utilized whole-genome sequencing to identify possible SNPs coding regions that could be responsible for the observed phenotypic suppression. We mapped and validated by PCR a missense mutation unique to *ΔmstA*-sup to an uncharacterized transcription factor, *ycjW*. The nucleotide substitution, G to A on the coding strand, results in an amino acid change from serine to asparagine at residue 258 (Fig. 2a). Sanger sequencing confirmed the presence of the same SNP in a second, independent, *ΔmstA*-sup isolate as well.

YcjW is annotated as a putative member of the LacI/GalR family of repressors that are largely responsible for carbohydrate metabolism. Common features of the family include an N-terminal helix-turn-helix DNA-binding domain, a linker domain, and a C-terminal ligand-binding domain^11^. To investigate SNP functionality, we constructed two strains, bearing a plasmid expressing either wild-type YcjW (pLLY1) or S258N YcjW (pLLSN1), in the background of *ΔmstA/ΔycjW*. Figure 2b shows that only plasmid-expressed mutated YcjW is able to restore H_2_S production, quantitated by utilizing the WSP5 probe, and qualitatively shown by lead acetate assay. Furthermore, only Δ*mstA*/Δ*ycjW*;P_LL_-*ycjW* (S258N) has an increased survival rate when challenged with gentamicin, H_2_O_2_, and nalidixic acid (Fig. 2c, d; Supplementary Fig. 2). Thus, we confirm that S258N YcjW in *ΔmstA*-sup is responsible for the increased hydrogen sulfide production and antibiotics and oxidative stress tolerance relative to *ΔmstA*.

### Genome-wide mapping of the YcjW regulon in vivo

**Fig. 3:**
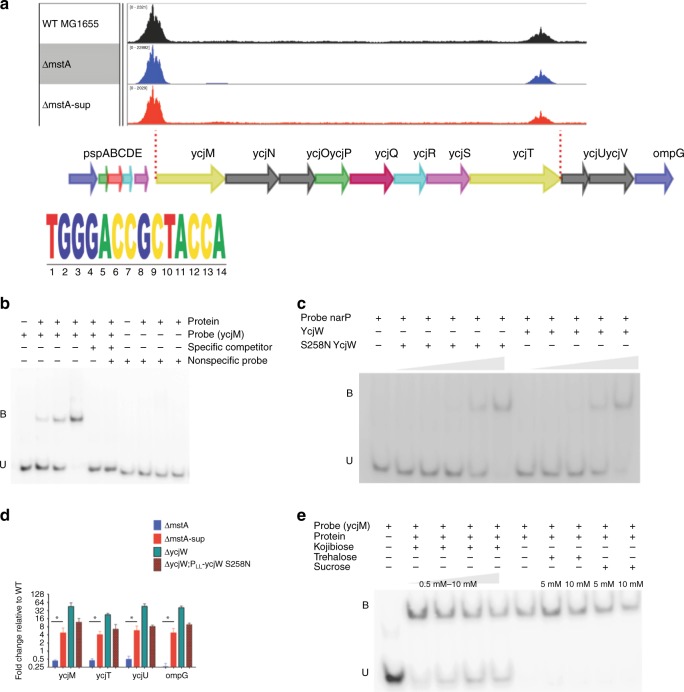
YcjW shows binding enrichment near *ycjM* and ycjU and regulates expression of operon *ycjMNOPQRSTUV-ompG*. **a** Represented on Integrative Genomics Viewer (IGV), are sorted, aligned sequences containing pileup data to reference genome NC_000913.3. MACS2 was used for peak calling. Enriched peaks are upstream of *ycjM* and *ycjU*. A 14 nucleootide sequence identified as the binding motif for YcjW. **b** YcjW protein was titrated to DNA:protein ratios of 1:0.5, 1:1, and 1:2. Unlabeled ycjM probe was added to the reaction in excess to compete for binding. Unbound (free) probe and YcjW-probe complexes are denoted as U and B, respectively. **c** YcjW and S258N YcjW proteins were titrated to DNA:protein ratios of 1:0.125, 1:0.25, 1:0.5, 1:1, and 1:2. with *narP* probe. Unbound (free) probe and YcjW-probe complexes are denoted as U and B, respectively. **d** qRT-PCR of a subset of genes in the *ycjM-*V and *ompG* operon. The absence of *ycjW* results in massive upregulation. *mstA-sup* and *ycjW;*P_LL—_ycjW both showed moderate and significant increased expression, while mRNA levels are repressed in *mstA*. Values are means ± SD (*n* = 3). *p < 0.05 as determined by the Student's *t* test. **e** YcjW protein was pre-incubated with Kojibiose, trehalose, or sucrose before radiolabeld DNA probes were added to the mixture. Only kojibiose prevented complex formation at a concentration of 0.5 mM. In contrast, both sucrose and trehalose added in excess at 5 mM and 10 mM failed to disrupt binding. Unbound (free) probe and YcjW-probe complexes are denoted as U and B, respectively. Source data are provided as a Source Data file

To identify transcriptional targets of YcjW, we performed ChIP-seq using an antibody against chromosomal 3xFLAG-tagged YcjW from wild-type, and *ΔmstA* cells, and 3xFLAG-tagged YcjW S258N from *ΔmstA*-sup cells. Figure 3a shows representative peaks identified by MACS2^12^ from aligned sequence reads. The most enriched regions, for all three strains, are at two sites near *ycjW*; the first site is before the translation start site of *ycjM* but after a predicted transcription start site, and the second lies between *ycjT* and *ycjU*. The binding motifs for LacI type family transcription factors are typically palindromes with a conserved central CG pair^13^. Recently, Zuo and Stormo experimentally tested the predicted binding motif for YcjW^14^. Combined with our analysis of peak summits, we found the same sequence in our data. Using the putative binding sequence, we further restricted peaks to ones containing the conserved 14 -bp motif, allowing up to three mismatches and with a fold enrichment greater than five. With those criteria, we identified two additional peaks specifically in *ΔmstA* that are not enriched in wild-type or *ΔmstA-sup*. The two sites are near the promoter of *narP*, encoding a two-component nitrate/nitrite response system and the other is located within the coding sequence of *cyaA*, encoding adenylate cyclase (Supplementary Fig. 3).

We then validated transcription factor binding through electrophoretic mobility shift assay (EMSA). We designed 50 -bp DNA probes containing the predicted binding sequence in the center. The YcjW protein reduced the mobility of the upstream *ycjM* DNA probe at about a 1:0.5 DNA:protein ratio. Increasing amounts of protein corresponded to an increase in YcjW–DNA complex (Fig. 3b). YcjW (S258N) also reduced DNA probe mobility at the same DNA:protein ratio, using *narP* probe (Fig. 3c). Titration of the normal protein and S258N YcjW showed that they both bound DNA probe starting at a DNA:protein ratio of 1:0.5. At a ratio of 1:2, no free DNA probe could be detected.

### YcjW is a repressor and S258N derepresses the YcjW regulon

The region downstream of *ycjM* contains a predicted operon consisting of ten genes-*ycjMNOPQRSTUV* and *ompG*. To test functionality of transcription factor-DNA binding to gene expression, we determined the amount of relative mRNA fold change using qRT-PCR. In Δ*ycjW*, representative genes, *ycjM, ycjT, ycjU*, and *ompG* are significantly upregulated, confirming that YcjW is a repressor (Fig. 3d). The absence of YcjW results in constitutive derepression of its regulatory transcriptional targets. The same genes exhibit a similar pattern, increased expression, in *ΔycjW*;P_LL_-*ycjW* S258N relative to wild-type MG1655 but not to the extent of its isogenic parent, *ΔycjW*. Consistent with *ΔycjW*;P_LL_-*ycjW* S258N, those genes are also upregulated in *ΔmstA*-sup, but not *ΔmstA*, suggesting that S258N YcjW affects DNA occupancy in vivo but not necessarily in vitro. Mutational analyses of other LacI-TFs have demonstrated that a single amino acid change in the C-terminal can alter effector or co-repressor binding and therefore DNA affinity at target sites^15,16^. The SNP is located in the C-terminal effector pocket of the protein, thus raising the possibility that it broadens specificity of inducer recognition, co-repressor binding affinity, or oligomerimerization. qRT-PCR of *narP* and *cyaA* showed no significant change in *ΔycjW*. A small subset of genes regulated by NarP was tested as downstream targets (Supplementary Fig. 4). Two genes, *nrfA* and *ydhU* did have a modest increase, while two others did not. NarP regulation, however, is complex and involves multiple regulators. Therefore, it is difficult to assess if YcjW-binding upstream of *narP* and *cyaA* is functional.

### Sugar kojibiose allosterically regulates the DNA-binding activity of YcjW in vitro

Because many LacI-type repressors act locally in response to some specific effector, we sought to identify the inducer for YcjW by considering its targets. YcjT is homologous to kojibiose phosphorylase from *Thermoanerobacter brockii* and *Pyrococcus* sp. Strain ST04^17,18^. Kojibiose phosphorylase can reversibly catabolize kojibiose to D-glucose and beta-D-glucose 1 phosphate. The downstream gene, *ycjU*, has experimentally been shown to encode a beta-phosphoglucomutase^19^. Again, utilizing EMSA, we tested to see if kojibiose is the effector molecule for YcjW. The addition of kojibiose at 1 mM disrupts the YcjW–DNA complex (Fig. 3e). Other disaccharides tested in excess of up to ten times, trehalose and sucrose, did not affect binding. However, attempts to grow *E. coli* K-12 MG1655 on minimal media with kojibiose as the sole carbon source were unsuccessful^20^. Growth on EZ Rich Defined media supplemented with kojibiose as the carbon source did grow but had a rather pronounced defect. Deletion of *ycjW* did not improve growth rates either (Supplementary Fig. 5). However, the concentration of kojibiose added to media is limited by its low solubility. It is possible that a higher concentration of kojibiose supplied would support enhanced growth. Taken together, our results indicate that kojibiose might not be the natural inducer of YcjW, but perhaps some derivative of kojibiose. Recently, the substrate for YcjM was identified as glucosylglycerate, alongside kojibiose for YcjT. Glucosylglycerate is an osmoprotectant in bacteria and archaea, and accumulates under salt stress and limited nitrogen availability^21,22^. However, most of our experiments were conducted in LB with amino acids constituting the main carbon source. We find it unlikely that synthesis of either the glycoside or disaccharide could occur without the appropriate substrate, and therefore is not likely involved in *ΔmstA* phenotypic suppression.

### Derepression of the YcjW regulon activates an alternative, PspE-dependent H_2_S synthesis pathway

While it is not directly evident how a cluster of carbohydrate catabolic genes regulated by YcjW can lead to an alternative pathway for H_2_S production, *pspE* encoding a thiosulfate sulfurtransferase, lies immediately upstream of *ycjM* and is transcribed in the same directionality. PspE, a rhodanense, has mercaptopyruvate sulfurtransferase activity, albeit low compared with thiosulfate^23^. It is part of a cluster of genes known as the phage-shock operon, consisting of *pspABCDE*. Although it can be coregulated with the other *psp* genes, it can also be transcribed independently from its own promoter^24^. PspF is the transcriptional activator and phage-shock protein A, encoded by *pspA*, negatively regulates PspF^25^.

**Fig. 4:**
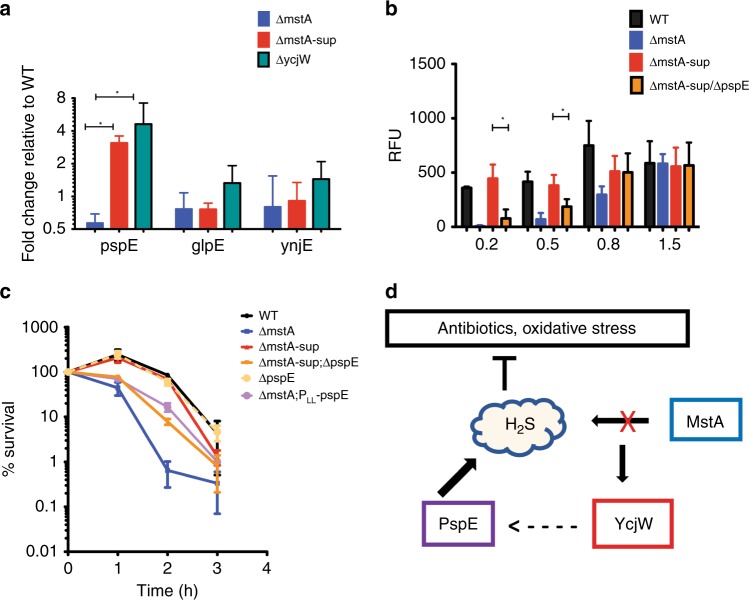
Deletion of *pspE* in Δ*mstA*-sup decreases H_2_S biosynthesis during exponential growth. **a** qRT-PCR of three thiosulfate sulfurtransferase genes. RNA was isolated from cells grown to OD_600_ ~ 0.4. Only *pspE* expression was significantly increased in both Δ*mstA*-sup and Δ*ycjW*. Values are means ± SD (*n* = 3). *p < 0.05 as determined by the Student's *t* test. **b** H_2_S levels in Δ*mstA*-sup; Δ*pspE* were undetectable till OD_600_ reached 0.8. Values are means ± SD (*n* = 3). *p < 0.05 as determined by the Student's t test. **c** Δ*mstA*-sup/Δ*pspE* had increased sensitivity to gentamicin compared with WT and Δ*mstA*-sup. Overexpression of pspE in Δ*mstA* increased tolerance to gentamicin compared with Δ*mstA*. Values are means ± SD (*n* = 3). **d** In cells lacking MstA which is responsible for H_2_S production, S258N YcjW upregulates PspE as an alternate route of H_2_S biosynthesis. Source data are provided as a Source Data file

*pspE* mRNA expression is significantly increased in Δ*mstA*-sup and Δ*ycjW* relative to wild-type cells. In contrast, there are no significant differences in relative expression of the other two thiosulfurtransferase genes (Fig. 4a). Furthermore, H_2_S production is undetectable during early exponential and mid-log phase in *ΔmstA*-sup/*ΔpspE* (Fig. 4b). However, at late logarithmic phase, H_2_S levels are now detectable to the same degree as MG1655 and *ΔmstA*-sup. In addition, overnight incubation with lead acetate strips shows no discernable difference in H_2_S extracellular production between the three strains (Supplementary Fig. 6). We conclude from the significant delay of H_2_S generation in *ΔmstA*-sup/*ΔpspE* that PspE is capable of generating H_2_S in early growth phases as observed in *ΔmstA-*sup. However, at later growth stages, another pathway for H_2_S production is activated and/or PspE is no longer sufficient. Moreover, *ΔmstA*-sup/*ΔpspE* also has increased sensitivity to gentamicin treatment compared with *ΔmstA-sup* and *E. coli* MG1655, but not quite as sensitive as *ΔmstA*. Overexpression of PspE in *ΔmstA* increases survival rate but only to the extent of *ΔmstA-sup/ΔpspE*, not *ΔmstA-sup* or wild-type (Fig. 4c). Altogether, we conclude that the SNP in *ycjW* resulted in increased expression of *pspE* in *ΔmstA*-sup. This is sufficient but not wholly responsible for increased H_2_S biosynthesis and in turn, the phenotypic suppression observed in *ΔmstA*-sup. We propose a model wherein, *E. coli* cells lacking 3MST acquire a SNP in transcription factor YcjW. The SNP imparts moderate constitutive expression of both YcjW targets and of *pspE*. Thiosulfate sulfurtransferase PspE is then able to increase H_2_S production in *ΔmstA*, and subsequently protect the cells from antibiotics and H_2_O_2_ induced stress (Fig. 4d).

The region upstream of *pspE* neither contains a strong binding motif for YcjW nor do the regions flanking regulators PspA and PspF. This is not entirely unexpected since none of the other genes tested in the *psp* operon were upregulated in *ΔmstA-*sup or *ΔycjW* (Supplementary Fig. 7). In addition, the moderate but significant increase of *pspE* mRNA in *ΔycjW*, in comparison with *ycjMNOPQRSTUV-ompG*, suggests a more complicated interaction than direct DNA binding. It may be indicative of “leaky” expression driven by the proximity of the 3′ end of *pspE* to predicted transcription start sites for *ycjM* and the binding site of YcjW.

Activation of *pspE* in the *ΔmstA-sup* with mutated *ycjW* mimics a natural physiological condition where the entire YcjW regulon is derepressed. Such condition is likely to be a change in carbohydrate availability, as most members of the YcjW regulon are predicted to function in carbohydrate metabolic pathways. Therefore, our results point to a link between changes in carbohydrate availability and PspE-dependent H_2_S production. The SNP in YcjW, regulating metabolism to at least two rare sugars, not only presents an interesting link between H_2_S and cysteine metabolism to carbon availability but also highlights the difficulty in studying a system in isolation. Pleiotropic phenotypes, especially should be considered within global cellular context. Moreover, a SNP in YcjW reflects the striking genetic plasticity employed by bacteria to promptly adapt to environmental changes and stimuli, and highlights the survival advantage imparted by endogenous H_2_S. Finally, both 3MST and rhodanese enzymes are found in the mitochondrion of human cells where they play an important role in sulfide oxidation pathways. Therefore, it is possible that the same interplay between the two types of enzymes, described here in a bacterial model, is highly evolutionary conserved and has implications in human health and disease^26,27^.

## Methods

### General growth conditions

For the general cultivation of *E. coli*, strains were grown in LB broth supplemented with 50 µg ml^−1^ kanamycin (cat. number 60615, Sigma), or 30 µg ml^−1^ chloramphenicol (cat. number C0378, Sigma) as appropriate. Growth on solid medium contained 1.5% agar added to LB. Where noted, MOPS EZ Rich Defined Medium Kit (cat. number M2105, Teknova) was used in place of LB^28^. Cysteine solutions were prepared immediately before use, as needed.

### Construction of strains and plasmids

For a list of all strains used throughout this work, refer to Table 1. BW25112 and its derivatives are from the *E. coli* Keio Knockout Collection (Thermo Scientific)^29^. Introduction of new mutations into *E. coli* MG1655 were achieved through P1 transduction as previously described^30^. Temperature-sensitive FLP recombinase plasmid pCP20 was used for the excision of selective markers, as needed^31^. All constructs were verified with PCR and sequencing. Primers used throughout this study are listed in Table S1.

**Table 1:** Strains used in this study

Strain name	Description	Source
*E. coli* K12 MG1655	F- lambda- *ilvG*- *rfb*-50 *rph*-1	^31, 32^
Δ*mstA*		^3^
Δ*mstA*-sup	Δ*mstA;ycjW*^S258N^	This study
Δ*mstA;*Δ*ycjW*		This study
Δ*mstA;*Δ*ycjW;*P_LL_-ycjW		This study
Δ*mstA;*Δ*ycjW;*P_LL_ycjW^S258N^		This study
Δ*ycjW*; P_LL_ycjW^S258N^		This study
WT-3xFlag	*E. coli* K12 MG1655 (ycjW fused to 3X FLAG)	This study
Δ*mstA-*3XFlag	Δ*mstA* (ycjW fused to 3X FLAG)	This study
Δ*mstA*-sup*-*3XFlag	Δ*mstA*; *ycjW*^S258N^ (*ycjW*^S258N^ fused to 3X FLAG)	This study
Δ*pspE*		This study
Δ*mstA-sup;* Δ*pspE*	Δ*mstA;ycjW*^S258N^; Δ*pspE*	This study
Δ*mstA;*P_LL_-pspE	Δ*mstA*; overexpression PspE	This study

To generate pLLY1, ycjW was PCR amplified from *E. coli* MG1655 using primers LL10 and LL11 and cloned into pACYC184 plasmid (NEB) using the Gibson Assembly Mastermix, according to the manufacturer’s protocol (NEB). Plasmid pLLSN3 was generated as above, except ycjW was PCR amplified from mstA-sup. The Q5 Site-Directed Mutagenesis Kit (NEB) was used to generate pLLSN1 from pLLY1, according to the manufacturer’s protocol.

Transformations were performed using the CaCl_2_ competent cell protocol^32^. All plasmids were sequenced for verification.

Addition of 3xFLAG tag to *ycjW* at its chromosomal locus was achieved as previously described, with slight modifications^33^. Briefly, primers pLL14 and pLL15 were used to PCR amplify Cm^R^ cassette from pKD4. PCR product was transformed into appropriate electrocompetent strains.

### H_2_S detection

End-point detection of H_2_S production by lead acetate strips (cat. number WHA2602501A, Sigma) were performed, as previously described^3^. Monitoring H_2_S generation with the WSP5 fluorescent probe (cat. number 1593024–78–2, Cayman chemicals) followed a modified protocol from Peng et al.^10^. Briefly, cells were grown in LB at 37 ^o^C to desired OD_600_ and aliquots of ~4 × 10^8^ cells were taken. The extinction coefficient used for calculations is OD_600_ of 1.0 is equal to 8 × 10^8^ cells. A working solution of WSP5 was made immediately before use, and added to cells for a final concentration of 10 µM. Samples were incubated at 37 ^o^C for 30 min and then washed in PBS buffer, pH 7.4, to remove excess probe. Cells were resuspended in PBS buffer and incubated at room temperature for 30 min. Cytation3 (Biotek) was used to take fluorescent readings, at excitation 500 nm and emission 533 nm. All experiments were repeated for a total of three times. Background subtracted values were normalized to cell number during analysis.

### Time-kill assay and growth curves

Overnight cultures of *E. coli* were diluted 1:300 into fresh media and grown to an OD_600_ of ~0.2. A 1 -ml aliquot was serially diluted and plated onto LB agar plates to determine initial colony-forming units per ml (c.f.u. ml^−1^) after overnight incubation at 37 ^o^C. Antibiotics were added to the cultures at indicated concentrations. Aliquots of 1 ml were collected at specified time intervals, serially diluted and plated. The results from three independent experiments were plotted in GraphPad version 5.0.

Growth curves were generated from Bioscreen C automated growth analysis system as previously described^3^. Antibiotics were purchased from Sigma-Aldrich or Gold Biotechnology.

### Whole-genome sequencing

Overnight cultures of *E. coli* cells were used for genomic DNA isolation. The MasterPure Complete DNA Purification Kit (Epicentre) was used to purify DNA according to the manufacturer’s protocol. DNA samples were quantified using the Quant-IT PicoGreen dsDNA assay kit (Thermo Fisher) according to the manufacturer’s protocol. DNA was sheared to appropriate size with Covaris, followed by adaptor ligation. Sequencing was performed at New York University School of Medicine’s Genome Technology Center.

### Quantitative RT-PCR

Cells were grown until appropriate OD_600_, and aliquots were collected and treated with RNAprotect Bacteria Reagent (Qiagen). After 5 min, cells were harvested and resuspended in lysis buffer (RNase-free TE buffer, 10 mg ml^−1^ lysozyme, 100 µg ml^−1^). Trizol LS (Thermo Scientific) was used according to the manufacturer’s protocol to extract the total RNA. Samples were treated with DNase (Invitrogen) and purified using spin columns (Zymo Research). Superscript III reverse transcriptase (Invitrogen) was used to synthesize cDNA. qPCR reactions were amplified using Power SYBr Green PCR Master Mix (Applied Biosystems) with appropriate primer sets and the cDNA template.

### ChIP-seq

ChIP was carried out as previously described with the following modifications^34^. Briefly, cells were grown at 37 ^o^C to OD_600_ ~0.4 and a final concentration of 1% formaldehyde was added for in vivo cross-linking of nucleoprotein. A final concentration of 0.5 M glycine was added to the culture to quench the reaction after a 20 -min incubation. Cells were collected by centrifugation and washing twice with 1× cold Tris-buffered saline then frozen in liquid nitrogen and stored at −80^ o^C. Cells were resuspended in lysis buffer (50 mM Tris [pH 7.5], 100 mM NaCl, 1 mM EDTA, protease inhibitor [Roche], 10 mg ml^−1^ lysozyme). After incubation at 37 ^o^C, IP buffer (50 mM HEPES-KOH, 150 mM NaCl, 1 mM EDTA, 1% Triton X100, 0.1% sodium deoxycholate, 0.1% SDS, protease inhibitor [Roche]) was added at a 1:3 ratio. DNA was sheared using ultrasonicator Covaris M220 on a 10 s on and 10 s off cycle for a total of 50 cycles.

The supernatant was incubated with 3xFLAG antibody (Biolegend) and Dynabeads Protein G (Thermo Scientific) overnight at 4 ^o^C. Samples were then washed twice with IP buffer, once with IP buffer + 500 mM NaCl, once with wash buffer (10 mM Tris, 250 mM LiCl, 1 mM EDTA, 0.5% NP-40, 0.5% sodium deoxycholate), and a final wash with TE. Immunoprecipitated complexes were eluted in elution buffer (50 mM Tris, 10 mM EDTA, 1% SDS) at 65 ^o^C for 20 min. Samples were treated with RNAse A (Qiagen), at 42 ^o^C and then uncross-linked with elution buffer + pronase for 2 h at 42 ^o^C, followed by 6 h at 65 ^o^C. DNA was purified using ChIP Clean and Concentrate (Zymo Research). Prior to sequencing, DNA was checked on TapeStation 2200 for appropriate size (Agilent). ChIP experiments were repeated for a total of three replicates.

For sequencing, sample libraries were prepared by using the NEBNext ChIP-seq Library (Illumina), according to the manufacturer’s protocol. Samples were sequenced on NextSeq 500 (Illumina). Bowtie and MACS2 were used for aligning and peak calling, respectively^12^.

### Electrophoretic mobility shift assay

*Protein purification*: YcjW and S258N YcjW were cloned into plasmid pet28-SUMO using the Gibson Assembly Mastermix kit, according to the manufacturer’s protocol (NEB). Auto-induction media was used for maximizing protein yield^35^. Cells were harvested and resuspended in lysis buffer (1 M NaCl, 5 mM imidaziole, 5% glycerol, protease inhibitor cocktail [Roche]) and sonicated. AKTA Start system was used for chromatography with HisTrapHP columns (GE Healthcare Life Sciences). Columns were washed in wash buffer (50 mM Tris-Cl [pH 8.0], 10 mM imidazole, 5% glycerol, 500 mM NaCl), followed by gradient elution with elution buffer (50 mM Tris-Cl [pH 8.0], 250 mM imidazole, 5% glycerol, 250 mM NaCl). The SUMO tag was cleaved with SUMO protease in dialysis buffer (200 mM NaCl, 50 mM Tris-Cl [pH 8.0], 5% glycerol, 1 mM DTT). Samples were applied to a HiTrap HeparinHP column (GE). Columns were washed with buffer (20 mM Tris [pH 8.0]. In all, 50 mM NaCl, 5% glycerol), and eluted in elution buffer (20 mM Tris [pH 8.0], 1.5 M NaCl, 5% glycerol). The sample was concentrated to 5 mL and injected onto a Superdex 200 column with GF buffer (20 mM Tris-Cl [pH 8.0], 50 mM NaCl, 1 mM DTT).

*EMSA*: dsDNA probes containing the binding sequence were radiolabeled with gamma ^32^P rATP using T4 polynucleotide kinase (NEB). Labeled probes were purified by passage through size-exclusion columns (Bio-Rad). Binding reactions were done as previously described^14^. The gel was then exposed to a phosphor screen and visualized on Storm 820 Phosphorimager (GE Healthcare). Experiments with various disaccharides were done in a similar fashion, except purified protein was incubated with appropriate sugar for 20 min at room temperature before addition of radiolabeled probe.

## Supplementary information

Supplementary Information

Source Data

## Data Availability

Data underlying Figs 1, 2, 3, 4, and Supplementary Figs 4 and 7 are provided as Source Data files. All other data are available from the corresponding author upon reasonable request. All sequencing data that support the findings of this study have been deposited in NCBI SRA with the accession code PRJNA542143.

## References

[1] Szabo C (2018). A timeline of hydrogen sulfide (H_2_S) research: from environmental toxin to biological mediator. Biochem. Pharmacol..

[2] Kimura H (2015). Signaling molecules: hydrogen sulfide and polysulfide. Antioxid. Redox Signal..

[3] Shatalin K, Shatalina E, Mironov A, Nudler E (2011). H2S: a universal defense against antibiotics in bacteria. Science.

[4] Mironov A (2017). Mechanism of H2S-mediated protection against oxidative stress in *Escherichia coli*. Proc. Natl. Acad. Sci. U. S. A.

[5] Shukla P (2017). ‘On demand’ redox buffering by H_2_S contributes to antibiotic resistance revealed by a bacteria-specific H2S donor. Chem. Sci..

[6] Nzungize, L. et al. Mycobacterium tuberculosis metC (Rv3340) derived hydrogen sulphide conferring bacteria stress survival. *J. Drug Target.* 1–13. 10.1080/1061186x.2019.1579820 (2019). 10.1080/1061186X.2019.1579820 30730218

[7] Park S, Imlay JA (2003). High levels of intracellular cysteine promote oxidative DNA damage by driving the fenton reaction. J. Bacteriol..

[8] Toliver-Kinsky, T. et al. H_2_S, a bacterial defense mechanism against the host immune response. Infect. Immun. **87**, e00272–18 (2019). 10.1128/IAI.00272-18 PMC6300618 30323021

[9] Luhachack L, Nudler E (2014). Bacterial gasotransmitters: an innate defense against antibiotics. Curr. Opin. Microbiol..

[10] Peng B (2014). Fluorescent probes based on nucleophilic substitution-cyclization for hydrogen sulfide detection and bioimaging. Chem. Weinh. Bergstr. Ger..

[11] Fukami-Kobayashi K, Tateno Y, Nishikawa K (2003). Parallel evolution of ligand specificity between LacI/GalR family repressors and periplasmic sugar-binding proteins. Mol. Biol. Evol..

[12] Feng J, Liu T, Qin B, Zhang Y, Liu XS (2012). Identifying ChIP-seq enrichment using MACS. Nat. Protoc..

[13] Ravcheev DA (2014). Comparative genomics and evolution of regulons of the LacI-family transcription factors. Front. Microbiol..

[14] Zuo Z, Stormo GD (2014). High-resolution specificity from DNA sequencing highlights alternative modes of Lac repressor binding. Genetics.

[15] Hall BG, Betts PW, Wootton JC (1989). DNA sequence analysis of artificially evolved ebg enzyme and ebg repressor genes. Genetics.

[16] Lu F, Brennan RG, Zalkin H (1998). Escherichia coli purine repressor: key residues for the allosteric transition between active and inactive conformations and for interdomain signaling. Biochemistry.

[17] Yamamoto T (2004). Cloning and sequencing of kojibiose phosphorylase gene from Thermoanaerobacter brockii ATCC35047. J. Biosci. Bioeng..

[18] Jung J-H, Seo D-H, Holden JF, Park C-S (2014). Identification and characterization of an archaeal kojibiose catabolic pathway in the hyperthermophilic Pyrococcus sp. strain ST04. J. Bacteriol..

[19] Kuznetsova E (2006). Genome-wide analysis of substrate specificities of the Escherichia coli haloacid dehalogenase-like phosphatase family. J. Biol. Chem..

[20] Mukherjee K, Narindoshvili T, Raushel FM (2018). Discovery of a kojibiose phosphorylase in Escherichia coli K-12. Biochemistry.

[21] Franceus, J., Pinel, D. & Desmet, T. Glucosylglycerate phosphorylase, an enzyme with novel specificity involved in compatible solute metabolism. *Appl. Environ. Microbiol*. **83**, e01434–17 (2017). 10.1128/AEM.01434-17 PMC5601347 28754708

[22] Klähn S, Steglich C, Hess WR, Hagemann M (2010). Glucosylglycerate: a secondary compatible solute common to marine cyanobacteria from nitrogen-poor environments. Environ. Microbiol.

[23] Cheng H, Donahue JL, Battle SE, Ray WK, Larson TJ (2008). Biochemical and genetic characterization of PspE and GlpE, two single-domain sulfurtransferases of Escherichia coli. Open Microbiol. J..

[24] Brissette JL, Weiner L, Ripmaster TL, Model P (1991). Characterization and sequence of the Escherichia coli stress-induced psp operon. J. Mol. Biol..

[25] Jovanovic G (2014). The N-terminal amphipathic helices determine regulatory and effector functions of phage shock protein A (PspA) in Escherichia coli. J. Mol. Biol..

[26] Mishanina TV, Libiad M, Banerjee R (2015). Biogenesis of reactive sulfur species for signaling by hydrogen sulfide oxidation pathways. Nat. Chem. Biol..

[27] Filipovic MR, Zivanovic J, Alvarez B, Banerjee R (2018). Chemical biology of H_2_S signaling through persulfidation. Chem. Rev..

[28] Neidhardt FC, Bloch PL, Smith DF (1974). Culture medium for enterobacteria. J. Bacteriol..

[29] Baba T (2006). Construction of Escherichia coli K-12 in-frame, single-gene knockout mutants: the Keio collection. Mol. Syst. Biol..

[30] Thomason, L. C., Costantino, N. & Court, D. L. E. coli genome manipulation by P1 transduction. *Curr. Protoc. Mol. Biol.* **Chapter 1**, Unit 1.17 (2007). 10.1002/0471142727.mb0117s79 18265391

[31] Datsenko KA, Wanner BL (2000). One-step inactivation of chromosomal genes in Escherichia coli K-12 using PCR products. Proc. Natl Acad. Sci. USA.

[32] Seidman CE, Struhl K (2001). Introduction of plasmid DNA into cells. Curr. Protoc. Protein Sci. Append..

[33] Uzzau S, Figueroa-Bossi N, Rubino S, Bossi L (2001). Epitope tagging of chromosomal genes in Salmonella. Proc. Natl Acad. Sci. USA.

[34] Grainger DC (2004). Genomic studies with Escherichia coli MelR protein: applications of chromatin immunoprecipitation and microarrays. J. Bacteriol..

[35] Studier FW (2005). Protein production by auto-induction in high density shaking cultures. Protein Expr. Purif..

