# Supplementary material for: Transcription factor YcjW controls the emergency H2S production in E. coli
Source: Nat Commun. 2019 Jun 28;10:2868. doi: 10.1038/s41467-019-10785-x (PMC6599011; doi:10.1038/s41467-019-10785-x)
Supplement: Supplementary file 1 — Supplementary Information [file 41467_2019_10785_MOESM1_ESM.docx]

**Supplementary information**

**Transcription factor YcjW controls the emergency H_2_S production in *E. coli***

Lyly Luhachack^1^, Aviram Rasouly^1,2^, Ilya Shamovsky^1^, and Evgeny Nudler^1,2^

1 Department of Biochemistry and Molecular Pharmacology, New York University School of Medicine, New York, NY, 10016, USA

2 Howard Hughes Medical Institute, New York University School of Medicine, New York, NY, 10016, USA. evgeny.nudler@nyumc.org


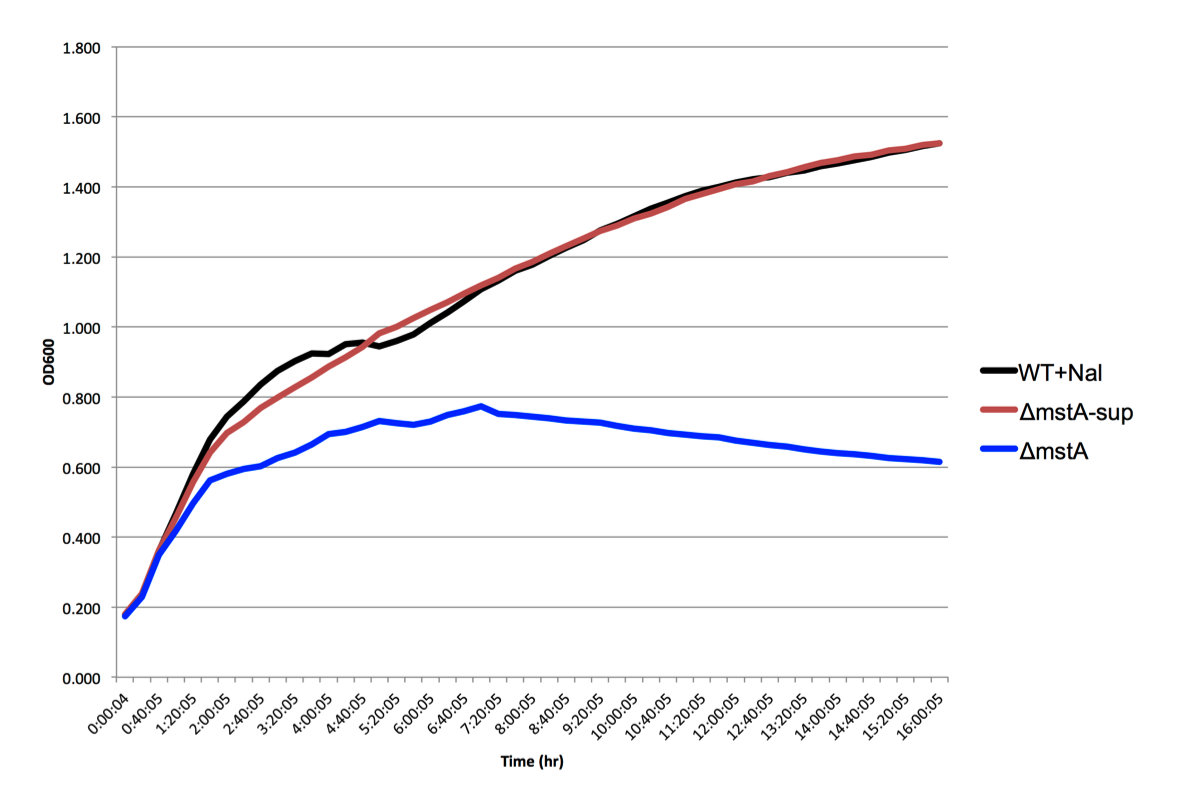


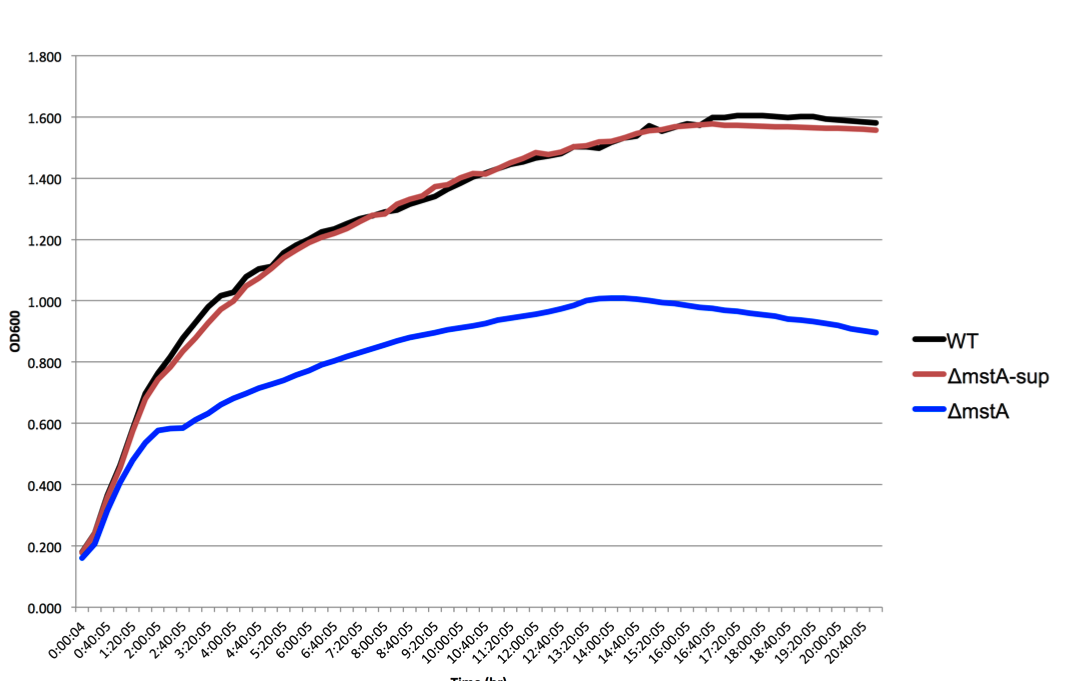


**Supplementary Figure 1.** Δ*mstA*-sup has increased tolerance to carbenicillin and nalidixic acid. **(a)** Cells were grown in the presence of 2ug ml^-1^ nalidixic acid and monitored for growth by OD_600_. Δ*mstA*-sup grows as well as wild type **(b)** Cells were grown in the presence of 1ug ml^-1^ carbenicillin and monitored for growth by OD_600_. Δ*mstA*-sup grows as well as wild type.


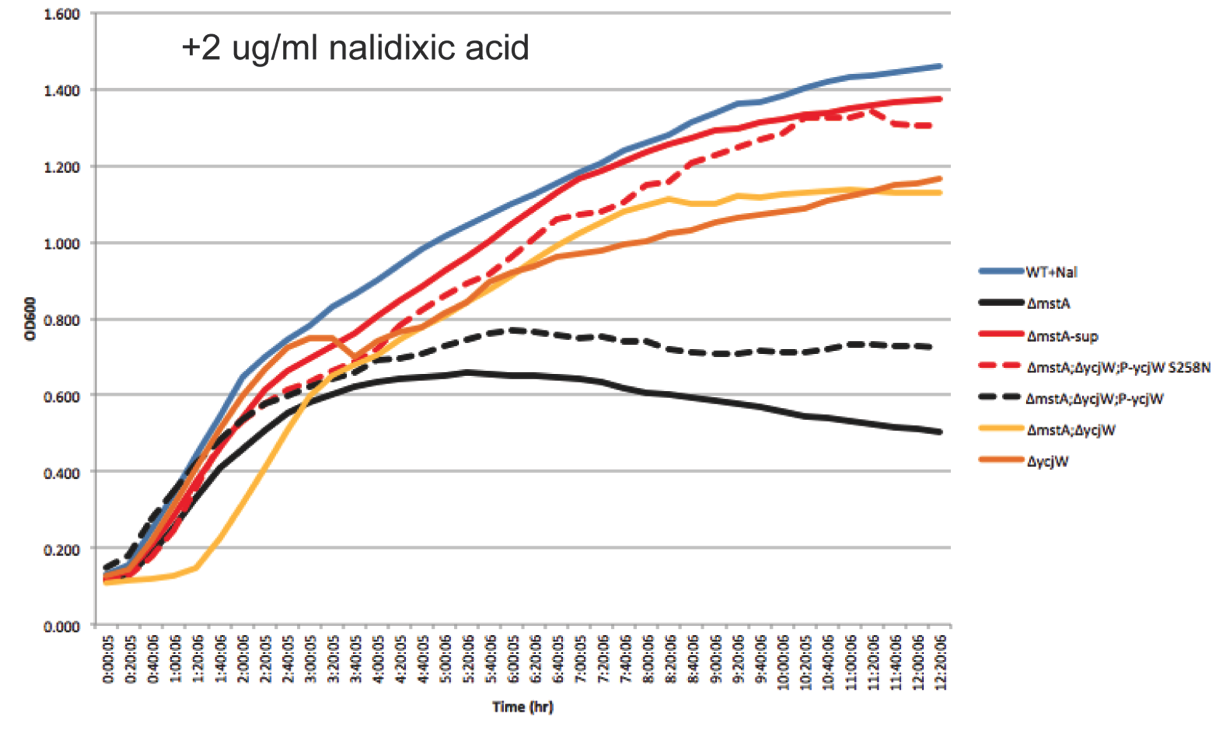


**Supplementary Figure 2.** Different strains of Δ*ycjW* and Δ*mstA* mutant combinations, grown in the presence of nalidixic acid. *ΔmstA***/***ΔycW*; P_LL_-ycjW S258N has similar growth rate to WT and *ΔmstA-*sup when grown in the presence of a sublethal concentration of nalidixic acid. In contrast, *ΔmstA***/***ΔycW*; P_LL_-ycjW has a reduced growth rate compared to wild type and *ΔmstA*-sup. Interestingly, *ΔycjW* also has impaired growth, though not to the extent of *ΔmstA*.


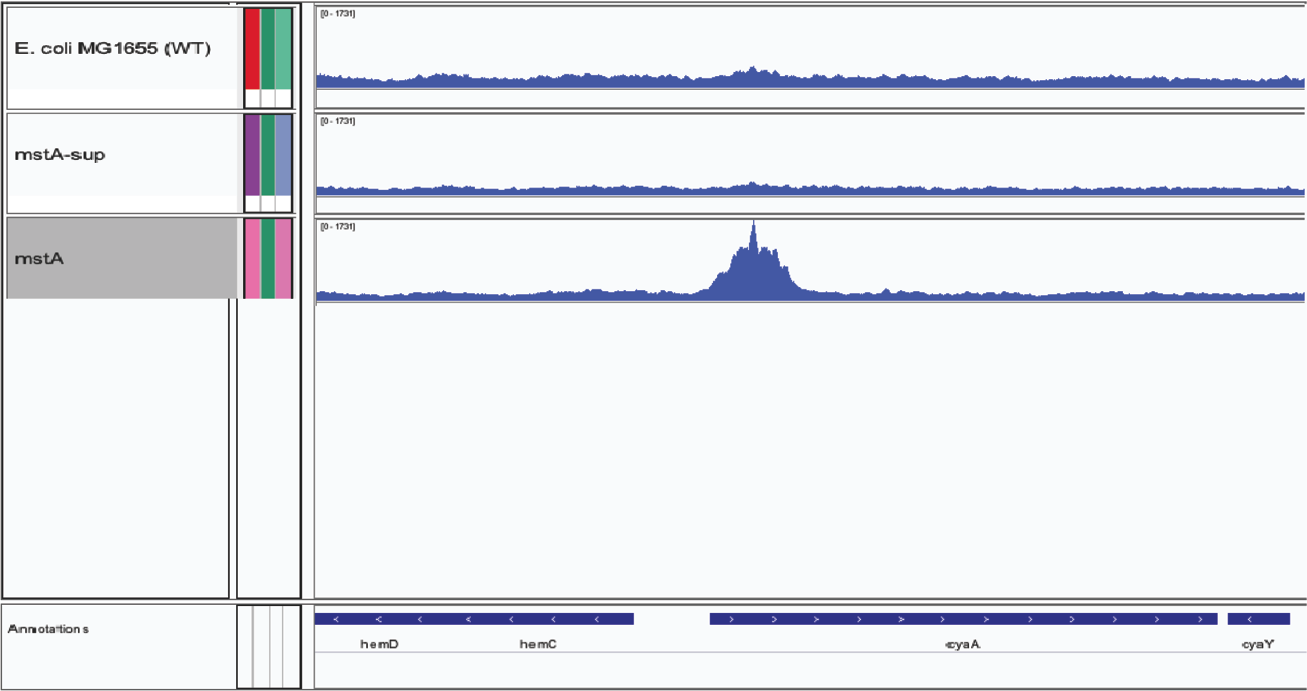


**Supplementary Figure 3.** ChIP-seq aligned data and pileup data visualized on IGV. Shown is the region near *cyaA*. Only *ΔmstA* showed an enrichment of binding at a site downstream of the translation start site of *cyaA.*


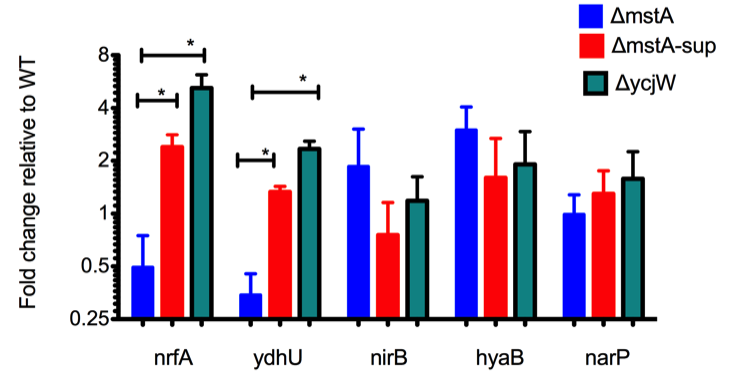

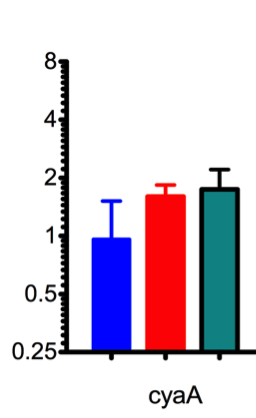


**Supplementary Figure 4.** YcjW does not affect *narP* expression or *cyaA*. NarP is a two-component nitrate/nitrite response regulator and can either repress or activate transcription. Of the genes measured for changes in mRNA levels, only *nrfA* and *ydhU* are significantly increased in *ΔycW*. Upregulation, however, is modest. Values are means±SD (n=3). * p<0.05 as determined by the student t-test.


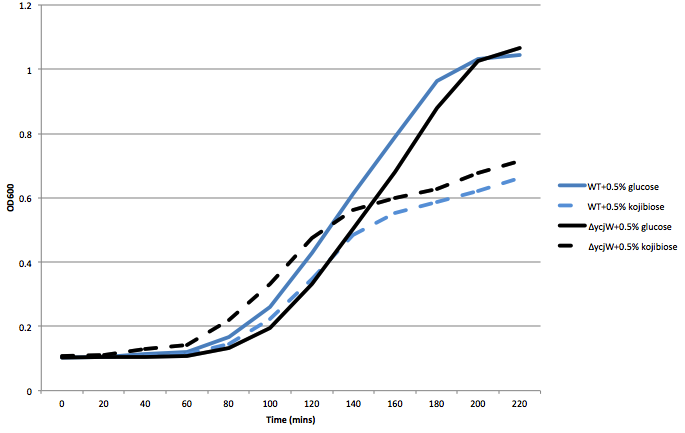


**Supplementary Figure Fig 5.** *E. coli* cells were grown in EZ rich defined media supplemented with either 0.5% glucose or 0.5% kojibiose as the carbon source. WT has a pronounced growth reduction with kojibiose. Knockout of *ycjW* did not improve growth.


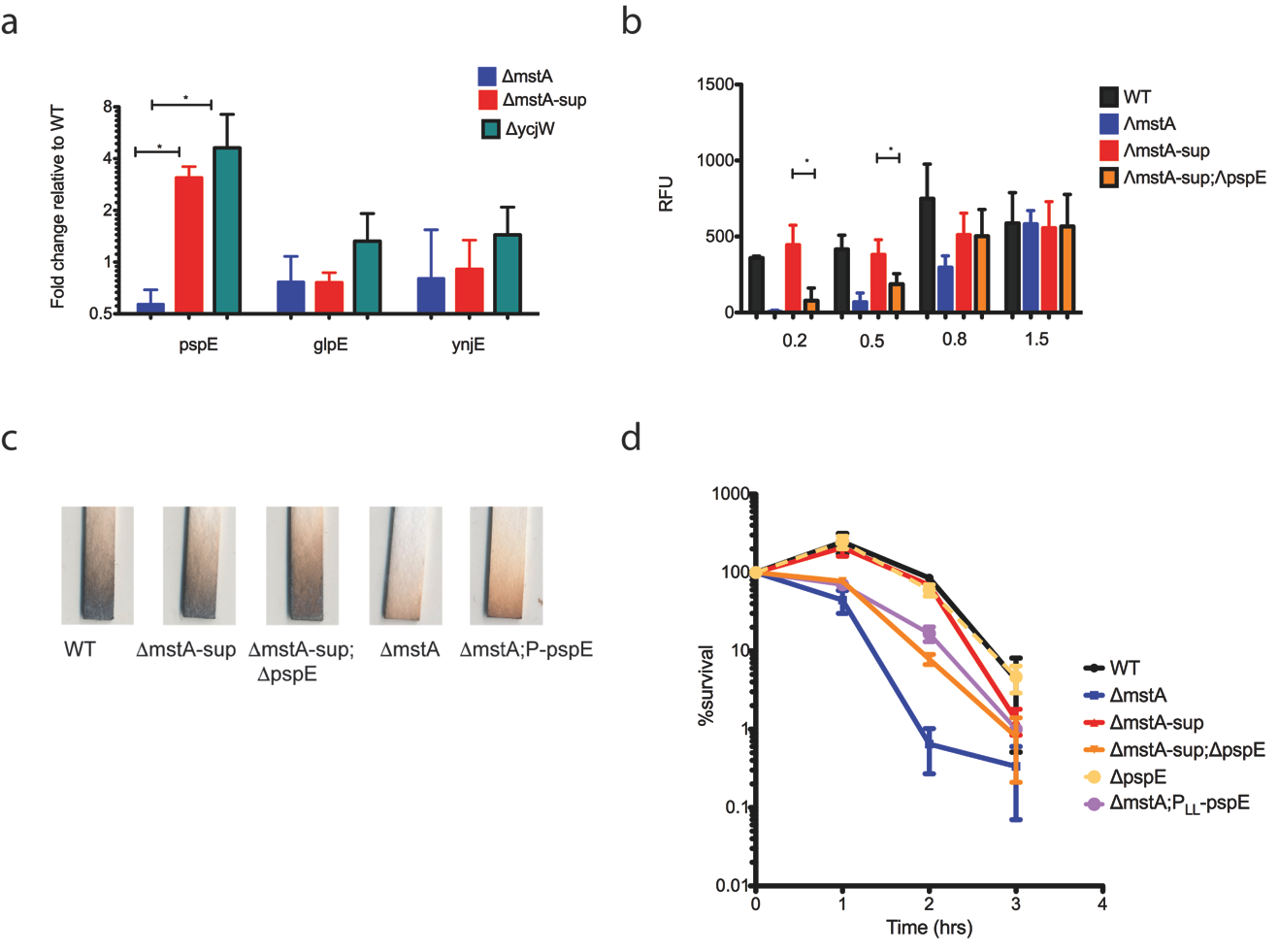


**Supplementary Figure 6.** After overnight incubation with lead acetate strips, *ΔmstA*-sup/*ΔpspE* is able to overcome the early deficit in H_2_S production seen during exponential growth. This is in contrast to *ΔmstA,* which does not accumulate extracellular H_2_S to the extent of WT after overnight growth.


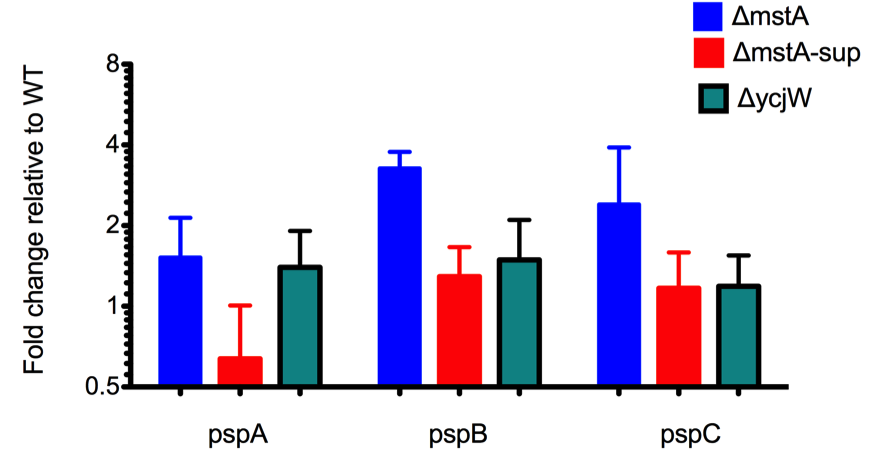


**Supplementary Figure 7.** YcjW does not affect other *psp* genes. *pspABC* is upstream of *pspE,* which is upregulated in *ΔycW,* indicating that the regulation of *pspE* in this case is distinct and separate from the phage shock operon in general. Values are means$\pm$SD (n=3).

**Table S1.** Primers used in this study.

| Name | Sequence | target | |
| --- | --- | --- | --- |
| LL01 | TTATACGCAAGGCGACAAGG | pKD3 CmR^27^ | |
| LL02 | GATCTTCCGTCACAGGTAGG | pKD3 CmR^27^ | |
| LL03 | CGGTGCCCTGAATGAACTGC | pKD4 KanR^27^ | |
| LL04 | CAGTCATAGCCGAATAGCCT | pKD4 KanR^27^ | |
| LL05 | CGGCCACAGTCGATGAATCC | pKD4 KanR^27^ | |
| LL06 | CACAATGGCCCGTTAGCAAC | mstA | |
| LL07 | TTTGCTGTGAAGCACCTGCG | mstA | |
| LL08 | AGTCGCTTCATTGCTGTCCT | | ycjW up |
| LL09 | CTTTCCGTTTCGCTGGAATA | | ycjW down |
| LL10 | gcataagggagagcgAGGCGCAGATGTCTGTCG | | pAC-ycjW |
| LL11 | gtagcccagcgcgtcTTATTTCAGCGTCATATGCG | | pAC-ycjW |
| LL12 | GAAATTAATGaacATTTATCGTGCGGCAGC | | pAC-ycjW SN |
| LL13 | AGGCTGTCAGTGGCAAAC | | pAC-ycjW SN |
| LL14 | CGGCGAACTCCATAAGATGT | | 3xFlag ycjW |
| LL15 | CTGGCATTCAGGCAAGGTAG | | 3xFlag ycjW |
| LL16 | CGACGTAAACTGTGCGGTAA | | glpE |
| LL17 | CGCCATGTAATCAACAAACG | | glpE |
| LL18 | GCA AAT GGG TTG AAG GAA AA | | cysB |
| LL19 | GCC GAA AAT AAC GCA AGA AA | | cysB |
| LL20 | TCGGGCTGCTAATAAACTGG | | pspE |
| LL21 | ATGTTGCCTTTCCGTGGTAG | | pspE |
| LL22 | AATGGATGGCAATTTTCATCCATAGAAGGACGCTTACATGGTGTAGGCTGGAGCTGCTTC | | pspE-pKD3 |
| LL23 | ACCTTTGACCTTCGGCATTGCGATGTCTTTCAGGCCACCGCATATGAATATCCTCCTTAG | | pspE-pKD3 |
| LL24 | GCCTTTAAAAAAGGCTTACTTGC | | pspE-AskA |
| LL25 | CCACCTTTGACCTTCGGCATTGC | | pspE-AskA |
| LL26 | CTAATGCCGGGTTACCTTGA | | cyaA-qRT |
| LL27 | GGCGGATCCTGTACTGACAT | | cyaA-qRT |
| LL28 | CGTGAACGTGAAATGTTTGG | | narP-qRT |
| LL29 | ATTCAACACCGAGGCAATCT | | narP-qRT |
| LL30 | CCGCAATAACGGTGAGTTTT | | ompG-qRT |
| LL31 | AAATCATGGCCTTCACGTTC | | ompG-qRT |
| LL44 | GATCAACTGGTTCACGCTCA | | nrfA-qRT |
| LL45 | CGTCCTGAATTGGCTTCATT | | nrfA-qRT |
| LL46 | TCGATGTTCGTGTTCCAGAG | | pspE-qRT |
| LL47 | ATCTCTTTTGCTTGCCCTGA | | pspE-qRT |
| LL48 | GCAGGCTTTCCATTTAACCA | | glpE-qRT |
| LL49 | GACCACATCGTAGCCCTGTT | | glpE-qRT |
| LL50 | ATCGTGAATGCCAACATCAA | | pspA-qRT |
| LL51 | TGTTCAATACGGCGAGTCAG | | pspA-qRT |
| LL52 | CGCGCTATTTCTGGCTATTC | | pspB-qRT |
| LL53 | TGCTCACTTTGCGACAATTC | | pspB-qRT |
| LL54 | AGCAGGCAAGCTGGTACTTC | | pspC-qRT |
| LL55 | CGGCGAGCAACTGATTTTAT | | pspC-qRT |
| LL56 | CGTGTTGTGAAATGTTGGGTTAA | | 16S |
| LL57 | CCGCTGGCAACAAAGGATA | | 16S |
| LL58 | GAGTAGTCTCTCGTTTCATGGGACCGCTACCACGGAAAGGCAACatgAAAC | | ycjM-EMSA |
| LL59 | GTTTcatGTTGCCTTTCCGTGGTAGCGGTCCCATGAAACGAGAGACTACTC | | ycjM-EMSA |
| LL60 | CTAATGCCGGGTTACCTTGATGGTAACGTTCCCAAAGGCATTTGCCTTTACACG | | cyaA-EMSA |
| LL61 | CGTGTAAAGGCAAATGCCTTTGGGAACGTTACCATCAAGGTAACCCGGCATTAG | | cyaA-EMSA |
| LL62 | GGTAACATTCACGCGCCTGGTAGCGTTACCAACGCTACGCTCAAACATAATGATTC | | narP-EMSA |
| LL63 | GAATCATTATGTTTGAGCGTAGCGTTGGTAACGCTACCAGGCGCGTGAATGTTACC | | narP-EMSA |
| LL64 | CCTGCCAATCAGGATCACGATACGCCGCTATTACAGCAATATTTTTCGtga | | nonspecific |
| LL65 | tcaCGAAAAATATTGCTGTAATAGCGGCGTATCGTGATCCTGATTGGCAGG | | nonspecific |
| LL66 | GTCGGCAAAAAGTGAATGGT | | ycjM-qRT |
| LL67 | GCATCTGGAATGGCGTTAAT | | ycjM-qRt |
| LL68 | CAACAGAAAACTGGCGTCAA | | ycjT-qRT |
| LL69 | CGCAGATGATAAAGCGCATA | | ycjT-qRT |
| LL70 | ACTGGCGTATCGCAAAATC | | ycjU-qRT |
| LL71 | GCATTCAGGGAGACAGAAGC | | ycjU-qRT |
| LL72 | CCGATGATCTGATGCTGGCT | | rpoD-qRT |
| LL73 | GTGCGCCCGATTTCAGATTC | | rpoD-qRT |
